# Evaluating Autoencoder-Based Featurization and Supervised Learning for Protein Decoy Selection

**DOI:** 10.3390/molecules25051146

**Published:** 2020-03-04

**Authors:** Fardina Fathmiul Alam, Taseef Rahman, Amarda Shehu

**Affiliations:** 1 Department of Computer Science, George Mason University, Fairfax, VA 22030, USA; falam5@gmu.edu (F.F.A.); trahman2@gmu.edu (T.R.); 2 Center for Advancing Human-Machine Partnerships, George Mason University, Fairfax, VA 22030, USA; 3 Department of Bioengineering, George Mason University, Fairfax, VA 22030, USA; 4 School of Systems Biology, George Mason University, Fairfax, VA 22030, USA

**Keywords:** protein modeling, tertiary structure, featurization, autoencoder, decoy selection

## Abstract

Rapid growth in molecular structure data is renewing interest in featurizing structure. Featurizations that retain information on biological activity are particularly sought for protein molecules, where decades of research have shown that indeed structure encodes function. Research on featurization of protein structure is active, but here we assess the promise of autoencoders. Motivated by rapid progress in neural network research, we investigate and evaluate autoencoders on yielding linear and nonlinear featurizations of protein tertiary structures. An additional reason we focus on autoencoders as the engine to obtain featurizations is the versatility of their architectures and the ease with which changes to architecture yield linear versus nonlinear features. While open-source neural network libraries, such as Keras, which we employ here, greatly facilitate constructing, training, and evaluating autoencoder architectures and conducting model search, autoencoders have not yet gained popularity in the structure biology community. Here we demonstrate their utility in a practical context. Employing autoencoder-based featurizations, we address the classic problem of decoy selection in protein structure prediction. Utilizing off-the-shelf supervised learning methods, we demonstrate that the featurizations are indeed meaningful and allow detecting active tertiary structures, thus opening the way for further avenues of research.

## 1. Introduction

Given the rapid pace with which wet and dry laboratories are generating molecular structure data, there is now a growing demand for machine learning (ML) methods to handle, summarize, and make observations from such data [1,2,3,4,5,6]. This is particularly true for proteins, where the strong relationship between three-dimensional (tertiary) structure and biological function [7] is spurring renewed interest in featurizing structure [8].

Research on featurizations of protein structure that retain information on biological activity is active. Such research falls primarily into two categories, one where researchers hand-engineer features and evaluate them in some function prediction task, and the other where statistical and ML methods are employed instead to discover such features. According to our knowledge, not one review can be found in the published literature to cover all such methods, but we point readers to the work in [9] on research in protein function prediction via feature engineering. Our focus in this paper is in the second category, as it removes the demands of acquiring domain-specific insight by researchers. Instead, statistical or ML methods promise to discover in a data-driven manner the pertinent features that summarize structure all the while retaining the functional information encoded in structure. Research on such methods shows varying performance. Among statistical methods, predominantly, linear, variance-maximizing methods, such as Principal Component Analysis (PCA) [10] have been favored due to their ease of implementation and evaluation [11,12,13]. Some work has also considered nonlinear methods [14], such as Isomap [15], Locally Linear Embedding [16], Diffusion Maps [17], and others [18]. Other work has considered topic models and has drawn observations regarding structure and function relationships in the universe of known protein structures [1]. The discovered features have been leveraged in important recognition tasks, such as predicting protein folds, function, and other properties [19], as well as in expediting the search for more structures and structural transitions of target proteins [20,21,22,23,24,25,26,27].

In the past decade, autoencoders (AEs) have gained popularity in the ML community for unsupervised feature learning [28,29]. AEs present highly versatile architectures that can be tuned to yield linear or nonlinear featurizations of data. Open-source neural network libraries, such as Keras, which we employ here, greatly facilitate constructing, training, and evaluating AE architectures and conducting model search.

AEs have yet to gain in popularity in the structure biology community, but some attempts have been made. The first occurrence can be found in [30], where an AE is applied to tertiary structures of a small molecule of 24 atoms. The presented AE is a deep one (as related in Section 2), but the risk of overfitting its numerous parameters in the presence of little data blunts the impact of this early work. In more recent work [31], a deep AE is applied to tertiary structures of two small molecules (one of 12 backbone dihedral angles, and another of 20 amino acids); the structures are collected from molecular dynamics simulations, and the goal is to reveal collective variables with which to expedite the sampling of more equilibrium structures. Work in [32] investigates a similar AE to summarize the folding landscape of Trp-Cage, a small polypeptide of 20 amino acids. Despite the focus being on small systems and on elucidating very specific properties of these systems, these applications of AEs motivate us to further consider and evaluate AEs for featurizations of tertiary structure data at scale.

Specifically, motivated by rapid progress in neural network research and some early adoption of AEs for analysis of molecular structures, we investigate and evaluate AEs yielding linear and nonlinear featurizations of protein tertiary structures. We build over preliminary published work [33], where we compare linear and nonlinear architectures. In this paper, we expand this analysis to more architectures that additionally allow incorporating external constraints on the sought features. We point to a best architecture for tertiary protein structures generated by template-free protein structure prediction methods. In addition, we demonstrate the utility of AEs in a practical context. Employing AE-based featurizations, we address the classic problem of decoy selection in protein structure prediction. Utilizing off-the-shelf supervised learning methods, we demonstrate that the featurizations are meaningful and allow detecting active tertiary structures, thus opening the way for further research on AEs and their utilization for structure–function studies of proteins and other molecular systems.

The rest of this paper proceeds as follows. Section 2 briefly relates some preliminaries and summarizes AEs. Section 3 describes the AE architectures we investigate, relates various details regarding training and evaluation, and describes in greater detail the utilization of AEs for the problem of decoy selection. Section 4 presents a detailed comparative evaluation of various AEs against a baseline linear model (PCA) on decoy data over a benchmark set of protein targets often used by decoy generation algorithms, as well as relates results on the decoy selection task. Section 5 concludes the paper with a discussion of future work.

## 2. Preliminaries

We do not aim to provide a detailed overview of AEs and their history in this paper. The interested reader is pointed to Refs. [29,34]. Here, we summarize preliminaries most pertinent to our study.

**Figure 1:**
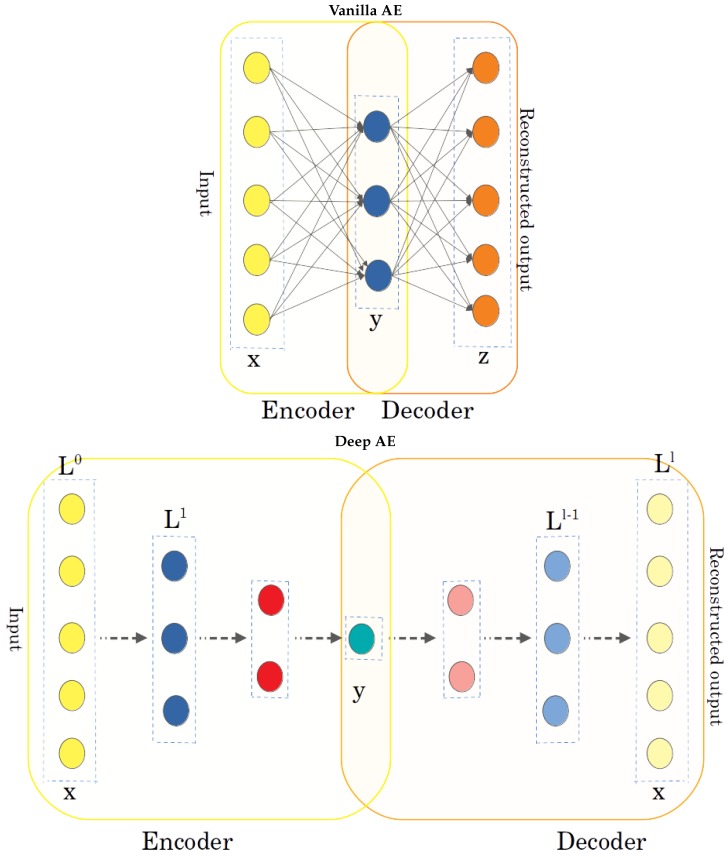
(**Top panel**): In a vanilla AE, the encoder maps *x* directly to *y*, and the decoder maps *y* directly to *z*. (**Bottom panel**): In a deep, non-stacked architecture, the encoder and decoder contain several hidden layers.

We begin by pointing out that all AEs contain an *encoder* and a *decoder*. Each contain one or more layers of neurons/units. The neurons in the first layer in the encoder are fed the elements of the input. The encoder *maps* the input layer *x* to its output layer *y*. The decoder mirrors the encoder and maps the same layer *y* to its output layer *z*. The layer *y* contains the learned code or reduced representation learned for the input *x*. The top panel in Figure 1 shows a vanilla AE, where a 4-dimensional input *x* is mapped to a 2-dimensional code *y*. The bottom panel shows a deep AE, where the encoder and decoder contain several hidden layers. An alternative architecture, which stacks vanilla AEs, has been investigated in [33]. We do not dwell on it here, as recent work in [33] shows that, while they converge faster than deep AEs, the quality of the reconstruction (described below) of tertiary structures is comparatively poor.

The *encoder* is a deterministic mapping $f_{\theta }$ parameterized by a vector of parameters θ={W,b} that transforms *x* into *y*. Typically, one seeks a *reduced* representation (|y|<|x|), and $f_{\theta }$ is an affine mapping that can be followed by a nonlinearity: $f_{\theta }\left(x\right)=\sigma \left(W\;\cdot \;x+b\right)$. Here, σ is the sigmoid function $\sigma \left(x\right)=\frac{1}{1+e^{-x}}$, and *W* and *b* are the weights and biases that connect neurons of one layer to those of another. The sigmoid is a specific activation function; there are many others. The *decoder* performs $z=g_{\theta^{{}^{\prime }}}\left(y\right)$, where $\theta^{{}^{\prime }}=\left\{W^{{}^{\prime }},b^{{}^{\prime }}\right\}$; $W^{{}^{\prime }}$ and $b^{{}^{\prime }}$ are the weights and biases, and $g_{\theta^{{}^{\prime }}}$ is an affine mapping followed (or not) by nonlinearity. The decoder seeks to reconstruct *x* via *z*.

AE Training: An AE learns *y* in a data-driven manner. The *training* of an AE is guided by a loss function that, for real-valued data, measures the reconstruction error $||x-{z||}^{2}$. Parameters θ and $\theta^{{}^{\prime }}$ are learned via gradient-based minimization of this error. The Adam optimizer [35] has been shown superior in many applications, including in our recent work [33]. Since the loss function may be high-dimensional, its optimization proceeds in epochs. In each epoch, the training data is divided into batches. In each epoch, parameters are updated after a batch is passed forward. The negative gradient of the loss function is evaluated and passed backwards to update the weights and biases.

Vanilla AEs: If there are no other layers between the input layer *x* and the code layer *y* (and, mirrorwise, the code layer *y* and the output layer *z*), one obtains a shallow/vanilla AE (shown in the top panel in Figure 1). We will refer to it as vAE. The number of weights (biases not included) in this shallow architecture is |x|×|y|×|z|. Since |x|=|z|, this number is ${\left|x\right|}^{2}\times \left|y\right|$. Typically, the desired |y| is low (not a function of |x|), so the number of weights is $O(|x{|}^{2})$. In a back-of-the-envelope calculation, when considering a molecule with 50 atoms, if *x* consists of the Cartesian coordinates of the CA atoms (the main carbon atom of each amino acid), then the number of weights in a vAE is 22,500.

Deep AEs: Deep AEs, to which we will refer as dAEs from now on, contain possibly many intermediate layers of different neurons (of typically decreasing number from the input layer to the code layer), as shown in the top panel in Figure 1. There are no prescriptions on the number of layers and the number of neurons per layer. Using the schematic in the bottom panel in Figure 1 as a reference, the number of weights that need to be learned is $|L^{0}{|}^{2}\times |L^{1}{|}^{2}\times \ldots \times |L^{l-1}{|}^{2}\times \left|L^{l}\right|$, with *l* denoting the number of layers *L* (0 being the input layer) and the decoder mirroring the encoder in its architecture; this number increases even further if one additionally considers the biases.

## 3. Methods

### 3.1. Number of Neurons Per Layers

The number of neurons in the code layer *y* shared between the encoder and decoder determine the desired dimensionality of the feature space. In this paper, expanding upon preliminary work in [33], we consider architectures where |y|∈{2,5,10,10,20}. Considering that tertiary structures occupy a Cartesian space of thousands or more dimensions, this is a drastic reduction in dimensionality.

### 3.2. Restricting Number of Layers

Depth comes at a cost, as it directly impacts the number of parameters (weights and biases) that have to be learned; equivalently, the dimensionality of the loss function/surface increases exponentially with increasing depth, which makes it particularly challenging for optimization algorithms to converge to a global minimum of the loss function. While data size in the structural biology community has steadily increased, it cannot approach the million regime available for image data. Significant computational resources are needed, for instance, to generate around 50–60K structures of a given protein sequence, as we do in this paper. Therefore, we consider AEs of limited depth.

Based on our preliminary evaluation in [33], which relates challenges with training very deep architectures, we restrict dAEs investigated here to only two intermediate hidden layers in the encoder and decoder. Specifically, we investigate the architecture $x=L^{0}\to L^{1}\to L^{2}\to y\to L^{2}\to L^{1}\to z$. While $|L^{2}|$ is much smaller than |x| but much larger than |y|, $L^{1}$ is chosen to be bigger than |x|, as this is shown to prevent overfitting and improve generalization [29]. Our preliminary work in [33] shows no overfitting for both vAE and dAE models.

#### 3.2.1. Regularization via Weight Tying

To further reduce the number of weights that have to be learned during training, we employ the so-called “weight-tying” trick. The weights of the decoder are not free parameters. Instead, $W_{\mathrm{decoder}}=W_{\mathrm{encoder}}^{T}$. This trick is a form of regularization, as it adds a constraint, thus reducing the dimensionality of the loss function surface.

#### 3.2.2. Regularization via Orthogonality

Alternatively, one can add an orthogonality constraint, where the weight matrices of the encoding and decoding layers are orthogonal to each other. This also means that we do not need to train the encoder and decoder separately, with the benefit of reduction of dimensionality of the loss function. To summarize, we have $W_{\mathrm{encoder}}$.$W_{\mathrm{decoder}}$=*I*, where *I* is the identity matrix.The same orthogonality constraint is enforced on all the intermediate encoding and decoding layers.

### 3.3. Activation Functions

One of the primary motivations for investigating AEs for featurizing tertiary protein structure data is their versatility in yielding linear versus nonlinear feature spaces via the choice of the activation function. We consider the following popular activation functions in the encoder and decoder: identity (I), sigmoid (σ), leaky RELU (LR), and parametric LR (PLR). Briefly, I(x)=x; σ(x) = $\frac{1}{1+e^{-}x}$; LR(x)=x, for x≥0 and αx otherwise; PLR turns α into a hyper-parameter learned during training. We note that we do not intend to exhaust all activation functions published in deep learning literature. With four options for the activation function in the encoder and decoder, this yields 16=4×4 different variants for each architecture considered (shallow versus deep). For instance, we refer to a vAE with sigmoid in the encoder but LR in the decoder as $vAE_{E_{\sigma },D_{LR}}$ and to a dAE with PLR in both the encoder and decoder as $dAE_{E_{PLR},D_{PLR}}$.

### 3.4. Exploring Model Space in Search of a Best Model

Considering the variation in the dimensionality of the code layer (y∈{2,5,10,20}), 16 combinations of activation functions, vAE versus dAE architectures, and architectures with or without the additional orthogonality constraint we design and train 128 different models. Each model is trained over training data and tested over testing data. The squared (reconstruction) error is measured over every instance in a testing dataset, and the mean of these values, the mean reconstruction error (MSE) is employed as a primary metric to evaluate a model. The MSE-based comparison related in Section 4 includes PCA as a baseline model, due to its popularity. In our Supplementary Materials, we investigated different AE architectures with different dimensionality, and the results clearly showed that, the dAE architecture $dAE_{E_{PLR},D_{PLR}}$ still dominates the performance. We note that due to its linearity, one can easily obtain the MSE for a PCA model. To keep the comparison in Section 4 fair, we “train” PCA over the same training dataset and “test” it over the same testing dataset as an AE model. The pca.fit and pca.transform function in Python’s sklearn library allow easily doing so; the pca.inverse_transform provides the MSE over a desired dataset.

#### Handling Non-Determinism

An AE model can converge to a different local minimum of the loss function during training. The optimization process depends on the initial values of the parameters, which are set at random. Therefore, we train each AE model 3 times (each time starting with random initial parameters), resulting in 3 trained variants. When evaluating a particular architecture, we relate the mean of the MSEs obtained over the 3 variants.

### 3.5. Interpreting the Learned Latent Features

The ability to interpret the learned features is valuable in protein studies. Typically, PCA is preferred, as the interpretation can easily be carried out over the axes of the latent space (the eigenvectors/principal components). A structure is selected as reference, and changes to it are introduced by deforming the structure along one latent axis while keeping the other constant. Visualization is then employed to note the type of structural changes encoded in the feature space.

We carry out a similar process here to visualize “walks” in the feature space. Specifically, we select a structure as reference. However, since one cannot deform a structure along an axis in a nonlinear space, we “hop” between structures whose encodings in the feature space are close to a line parallel to a selected axis. We show the corresponding structural changes in Section 4, providing insight into what information on structure variation is encoded in the latent dimensions of the feature space.

### 3.6. Supervised Learning over AE-Obtained Features

Beyond visualization of the latent feature space, a practical question concerns the utility of learned features for prediction tasks. In [33] we show that features learned with an AE can be used to in a supervised setting to predict the dissimilarity of a computed structure from an experimentally known, biologically active/native structure. Buoyed by these results and building over a more comprehensive model search in this paper, we evaluate the features learned in an unsupervised manner from the top AE model(s) to predict the lRMSD of a tertiary structure computed by a template-free structure prediction method from a known native structure. lRMSD refers to the popular least root-mean-squared-deviation metric [36]. The latter first finds an optimal superimposition of a decoy to a known native structure (extracted from the Protein Data Bank (PDB) [37]) to remove differences due to translation and rotation in 3D and then averages the Euclidean distance over the atoms.

For a target protein, the data at hand (over which we train and test AE architectures and models) consist of 50–60K tertiary structures generated with the Rosetta AbInitio protocol [38]. A proof-of-concept evaluation, which we carry out in [33], would be as follows. Split the AE-featurized data into a training and a testing dataset for a target protein, train a supervised learning method *on that protein*, and then evaluate the model on *that protein’s* testing dataset. We are not interested in such a task here. Instead, we consider the following, more general setting. Over a list of target proteins organized in three categories of difficulty (based on the quality of the tertiary structures generated for each protein), we select half the proteins in each category to constitute the training dataset and the rest to constitute the testing dataset. This setting is more realistic, as we build one model per category. We then consider yet another setting, where we build one model over all categories. We also show performance of supervised Learning over PCA features as well Isomap features which is a another nonlinear dimensionality reduction method.

### 3.7. Datasets, Implementation Details, and Experimental Setup

#### Data Collection

The evaluation is carried out on 18 proteins of varying lengths (53 to 146 amino acids long) and folds (α, β, α+β, and coil) that are used as a benchmark to evaluate structure prediction methods [39,40]. In an abuse of convention but in the interest of expediency, we refer to these proteins not by their actual names but by the PDB id of a representative native structure deposited for each of them in the PDB (Column 2 in Table 1). The names of the proteins can be found in the Supplementary Materials. On each target protein, we have run the Rosetta AbInitio protocol to obtain a dataset of no lower than 50,000 structures. The protocol takes as input an amino-acid sequence (in FASTA format) and a generated fragment library (which we have generated using the ROSETTA server). The protocol is run in an embarrassing parallel fashion, submitting batch jobs to our Mason ARGO super-computing cluster. The slight differences in the number of structures obtained for each protein are largely due to small variations in allotted time and increasing computational cost of the protocol to obtain all-atom structures for long sequences and/or complex folds.

Table 1 presents all the 18 proteins arranged into three different categories/levels of difficulty (easy, medium, and hard). These levels have been determined using the minimum lRMSD between Rosetta-generated decoys and a known native structure of the corresponding target protein (obtained from the PDB); the four-letter PDB ids are shown in Column 2; the fifth letter identifies the chain in a multi-chain PDB entry. These codes are used in an abuse of notation to refer to a particular protein (and its decoy dataset). The size of the dataset |Ω| for each target is shown in Column 5. Column 7 shows the percentage of native decoys within an lRMSD threshold of the known native structure; the values of these thresholds vary on the dataset and have been determined and related in prior work that focuses on clustering protein tertiary structures [42]. Column 7 relates the imbalance of the decoy datasets; in some cases, the near-native decoys constitute less than 5% of the dataset, which posits that decoy selection is a challenging ML problem.

#### Data Preparation

For each Rosetta-generated structure, we only retain its CA atoms. In each dataset, we designate a structure as the *reference* structure. We select this arbitrarily to be the first structure in a dataset. All structures are then optimally superimposed to the reference structure to minimize differences due to rigid-body motions [36] (that is, differences due to translations and rotations in 3D). The superimposition changes the coordinates of each structure except for the reference one. The reference structure is then subtracted from each superimposed structure to obtain atomic deviations (coordinate differences per atom). This “centralization” is common practice in how PCA is applied to a molecular structure data, and we follow the same process to prepare a dataset for training an AE model. Thus, the input fed to a model does not consist of atomic coordinates, but rather atomic deviations. It is, however, easy to obtain a reconstructed structure. Let us consider an input structure is *S*. After its superimposition to the reference structure, its atomic deviations from the input structure are dS. The encoder uses dS to obtain y(dS), which is the learned latent representation (the code). The decoder provides the reconstructed $\hat{dS}$. One can easily obtain the reconstructed structure $\hat{S}=\hat{dS}+S_{\mathrm{reference}}$.

The so-centralized dataset for each protein is split to obtain a training, validation, and testing dataset. A 0.5:0.1:0.4 split yields the training, validation, and testing datasets, respectively. We note that the performance over the validation dataset is monitored in tandem with the performance over the training dataset during training to ensure no overfitting or underfitting occurs.

#### Metrics of Model Performance

As summarized above, the squared (reconstruction) error is measured over every instance in a testing dataset, and the mean of these values, to which one refers as MSE, is a primary metric to evaluate a model. Specifically, the SE calculated on an instance corresponding to a structure *S* is $\left|\right|\hat{dS}-dS{\left|\right|}^{2}$; recall that the actual input to an AE (and to PCA) consists of atomic deviations corresponding to each structure. It is not hard to see that this evaluates to the same quantity as $\left|\right|\hat{dS}-dS{\left|\right|}^{2}$, as one can write that $\left|\right|\hat{dS}-{dS||}^{2}=\left|\right|\left(\hat{dS}+S_{\mathrm{reference}}\right)-\left(dS+S_{\mathrm{reference}}\right){\left|\right|}^{2}=\left|\right|\hat{S}-S{\left|\right|}^{2}$.

#### Implementation Details

We use Keras to implement, train and evaluate the various AEs investigated in this paper [43]; Keras is an open-source neural-network library written in Python. Each of the investigated AEs is trained for a total of 100 epochs with a batch size of 256. A learning rate of 0.008 is employed to prevent premature convergence to local optima. In [33], various dropout and learning rates are evaluated by hyper-parameter search. When the LR activation function is employed, the negative slope coefficient α is set to 0.3. Training times vary from 326.479 to 1076.575 s depending on the size of the training dataset. Since the proteins shown in Table 1 vary from 53 to 146 amino acids, input instances *x* vary in dimensionality from 159=53×3 to 438=146×3. So, in a vAE trained on tertiary structures of a protein of 53 amino acids, |x|=159. In a dAE, we set $|L^{\left(1\right)}|$ to 250 for all datasets where |x|<250, and to |x|+30 otherwise. The dimensionality of the second hidden layer is set to $|L^{\left(2\right)}|=125$.

## 4. Results

Our evaluation proceeds as follows. First, we visually show two-dimensional (2D) feature spaces obtained with PCA, Isomap, and some of the trained AE models on selected datasets. Then, we compare various AE architectures and trained models based on reconstruction error (measured via mean MSEs). We additionally show, what are the features obtained with the top AE model encode at a structural level. Finally, we employ those features for decoy selection, utilizing PCA- and Isomap-obtained features as the baselines for comparison.

### 4.1. Visualization of Learned Feature Spaces

**Figure 2:**
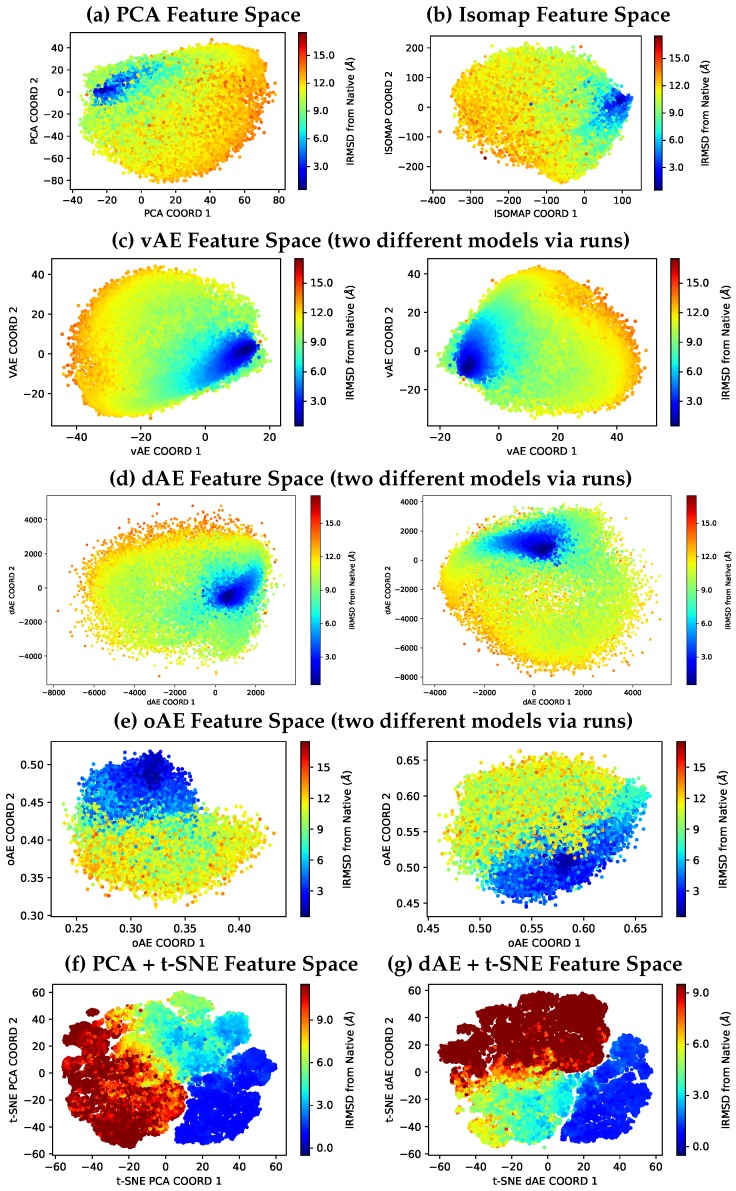
Tertiary structures obtained with Rosetta and prepared for input as described in Section 3 are projected onto the two features learned via (**a**) PCA, (**b**) Isomap, (**c**) best-performing vAE architecture, (**d**) best-performing dAE architecture, (**e**) best-performing oAE architecture, (**f**) PCA + t-SNE, or (**g**) best-performing dAE model + t-SNE; in (**c**–**e**), two feature spaces are shown to demonstrate the variability of model parameters converged onto from the learning process. (**a**–**g**) Featurized structures are drawn as disks, color-coded by their lRMSD (of the corresponding CA-represented structures) from the native structure in a blue-to-red color scheme indicating lower-to-higher lRMSDs.

We visualize the 2D latent space obtained by PCA, Isomap, the best-performing vAE model, the best-performing dAE model, and the best-performing oAE model. We do so on the Rosetta-obtained dataset for the protein with known native structure under PDB id 1dtd(B). In Figure 2, each tertiary structure is drawn as a disk whose coordinates are the features learned with the various models (PCA, Isomap, or AEs). In the case of AEs, these models are those with architectures, where the code layer *y* has 2 neurons.

Note that we are distinguishing between architecture and model. In a model, the parameters have been learned. Indeed, as related in Section 3, starting with parameters initialized at random, the same optimization process (using the Adam optimizer in our case) may lead to different near-optimal parameter values. We repeat this process 3 times for the AEs but relate in Figure 2 only projections that are different among the various AE models obtained for a particular AE architecture.

Figure 2 color-codes all featurized structures by their least RMSD (over CA atoms) from the native structure. The blue-to-red color-coding scheme associates low-to-high RMSD values. Figure 2a shows the feature space obtained via PCA; Figure 2b shows the feature space obtained via Isomap; Figure 2c juxtaposes two different feature spaces obtained via different vAE models via runs (the vAE model is a linear one that MSE-based analysis (shown below) indicates that it is a superior architecture to other vAEs). Figure 2d juxtaposes two different feature spaces obtained via different dAE models via runs (corresponding to the best dAE architecture, $dAE_{E_{PLR},D_{PLR}}$, as indicated by the MSE-based analysis). Figure 2e shows the feature space obtained via different oAE models via runs (corresponding to the best oAE architecture, ${\mathrm{oAE}}_{\sigma_{E},\sigma_{D}}$, as indicated by the MSE-based analysis).

It is common in the literature to employ the t-Distributed Stochastic Neighbor Embedding (t-SNE) technique [44] for visualization but not as the primary dimensionality reduction technique, because it better preserves distances among neighboring data points, t-SNE can result in better visualizations. However, due to its quadratic time and space complexity in the number of data points, it is can be resource draining and slow. Therefore, it is highly recommended to use another dimensionality reduction method first and then to employ t-SNE for visualization. We do so as follows. We first employ PCA or our best-performing dAE architecture ($dAE_{E_{PLR},D_{PLR}}$) to reduce the data dimensionality to 15 and then use t-SNE to further reduce the dimensionality to 2. The resulting visualizations are shown in Figure 2f–g. Figure 2f shows the PCA+t-SNE feature space, and Figure 2g shows the dAE+t-SNE feature space.

Figure 2f–g allow making two important observations. First, whether using PCA or dAE first, the t-SNE-aided visualizations are much more informative than the ones above (Figure 2a–e). There is better co-localization of structures with similar lRMSDs from the native structure. Second, the dAE+t-SNE approach results in slightly more uniform groups of structures; structures with higher lRMSDs are better co-localized.

Figure 2a–f shows that co-localization of low-lRMSD structures in the feature spaces obtained via different models, suggesting that the features do capture inherent structural information. However, the dAE models are the ones that result, visually at least, in better co-localization of low-lRMSD structures.

### 4.2. MSE-Based Comparison

We compare various models (with different combinations of activation functions and varying dimensionality of learned codes) based on their mean MSE over the testing dataset for each protein. We do not exhaustively show all AE models (which number in the hundreds, as described in Section 3 but restrict to top ones, using PCA as a baseline. Note that Isomap is excluded from the MSE-based evaluation, as no such values can be obtained from Isomap. Specifically, Table 2 shows the mean MSE obtained via PCA in Column 2, the mean and variance MSE obtained via linear vAE models (we recall that for each AE architecture, we may obtain three different models via runs depending on the parameters initialization) in Column 3, the mean and variance MSE obtained via the best non-linear vAE architecture in Column 4, the mean and variance MSE obtained via the best oAE architecture in Column 5, and the mean and variance MSE obtained via the best dAE architecture in Column 6. The best non-linear vAE architecture (obtained via variation of non-linear activation functions) is the one where a sigmoid is used in the encoder (${\mathrm{vAE}}_{\sigma_{E}}$), as demonstrated from our prior analysis in [33]. The best dAE architecture is the one with a PLR activation function in both the encoder and decoder ($dAE_{E_{PLR},D_{PLR}}$), and the best oAE architecture is the one with a sigmoid in both the encoder and decoder (${\mathrm{oAE}}_{\sigma_{E},\sigma_{D}}$); in the interest of space, we do not show here the performance of other dAE and oAE architectures, but only relate the best one for each.

Table 2 shows that compared to PCA, most AE models achieve similar or lower mean MSEs. Linear vAE architectures obtain similar MSEs as PCA, which reflects the knowledge that a linear vAE reproduces PCA [45]. Nonlinearity in the encoder of a vAE does not significantly improve performance over a linear vAE. In contrast, the oAEs and dAEs achieve overall better results (lower mean MSE), with very few exceptions. The dAE architecture, with the $dAE_{E_{PLR},D_{PLR}}$ model in particular, achieves the best performance. We note that Table 2 shows that variances are typically very low, ≤$1\times 10^{-10}$, reaching between [0.019,0.059] on only 4 of the datasets.

### 4.3. Interpretation of Latent Features via Walks in Latent Space

**Figure 3:**
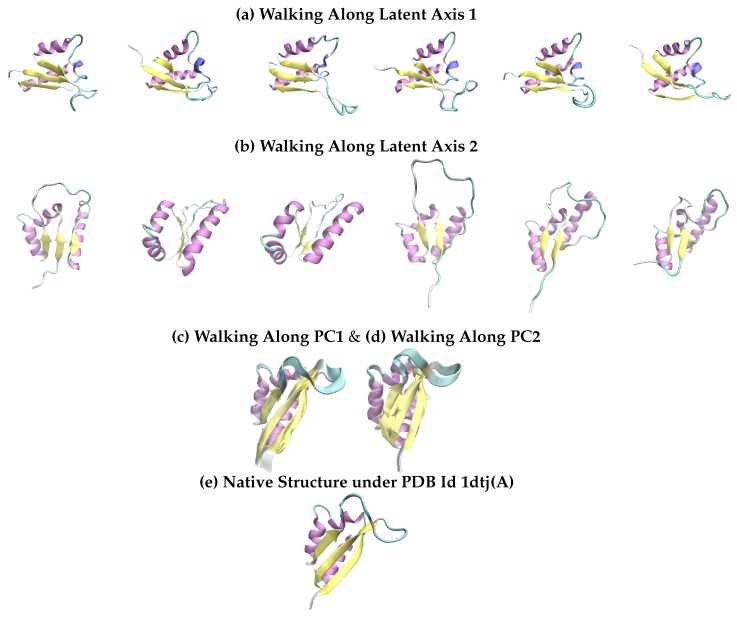
(**a**) The 6 drawn structures correspond to a horizontal, left-to-right walk in the latent space of PDB id 1dtj(A).(**b**) The 6 structures correspond to a vertical, bottom-to-top walk in the same latent space. They are shown from a different viewpoint to highlight structural changes. (**c**,**d**) 50 structures (generated via motions along each of the top two PCs) are superimposed to visualize the latent PC space. (**e**) The native structure is drawn for reference. (**a**–**e**) All structures are rendered with the VMD software [46].

We now investigate further the top-performing model, $dAE_{E_{PLR},D_{PLR}}$. In Figure 3 we provide further insight into the feature space learned by the $dAE_{E_{PLR},D_{PLR}}$ model trained to obtain 2D codes by visualizing “walks” in it. We focus on the protein Nova-2 KH3 K-homology RNA binding domain with known native structure under PDB Id 1dtj(A), which is shown in Figure 3e. This structure contains three β sheets (yellow-colored segments) in pairs of antiparallel sheets. The β sheets are separated by three α helices. Figure 3a draws 6 structures whose projections over the latent space lie on a directed, left-to-right segment (see Figure 2e for reference) almost horizontal to the first latent axis; that is, these structures have very similar values for their second latent feature. Conversely, Figure 3b draws 6 structures whose projections over the latent space lie on a directed, bottom-to-top segment almost horizontal to the second latent axis.

Interesting observations emerge. As expected, the latent features capture collective, non-local structural structural changes. Specifically, Figure 3a shows that changes in the first latent feature over the 6 shown structures concern primarily the packing between the β sheets and the α helices and affect the buried core of the protein. These are large motions (over 4Å) that additionally impact the orientation of the β sheets and the local structure of the loop connecting two of the β sheets. Figure 3b shows that changes in the second latent feature over the 6 shown structures are also large, collective motions (over 8Å) that impact the formation of at least one of the β sheets and the collective placement of the three sheets relative to the two α helices.

Extrapolating from these observations, it is reasonable to conclude that the two learned latent features control the bulk of the structural changes needed to obtain the sheets, and place them in the right orientation to one another and the alpha helices for a compact core. The latent features do not significantly impact the formation of the α helices. Indeed, the majority of the structures generated by Rosetta for this protein have the α helices already formed; this is known to be an easier task for fragment-based assembly methods in comparison to the challenging task of forming and packing β sheets; β sheets tend to have greater sequence separation [47].

To provide related information on what the walks along PC1 and PC2 capture, we can utilize the top two PCs directly as follows. The known native structure is “deformed” along a PC of interest per the equation $S_{\mathrm{reference}}+i\;\cdot \;\alpha \;\cdot \;\mathrm{PC}$, where *i* is varied in a range, and the parameter λ controls the deformation per step. We vary *i* as in −25≤i≤25 and set λ to 0.5. In this manner, we generate 50 structures that show the motion along PC1 and 50 structures that show the motion along PC2. These are shown in Figure 3c,d, respectively, superimposed over one another. As Figure 3c,d show, the motions encoded in PC1 and PC2 are localized and not as informative as walks along the AE coordinates.

### 4.4. Decoy Selection in AE-Learned Feature Space

To evaluate whether learned features encode information on nativeness, we evaluate learned features in the context of supervised learning. We focus on features learned via PCA, Isomap, and $dAE_{E_{PLR},D_{PLR}}$; we do not carry forward the vAE and oAE models, as the comparison above shows them to be inferior to dAE models. In each setting, we consider 2D, 5D, 10D and 20D feature spaces and carry out a regression task, as described in Section 3, where we aim to predict the lRMSD between a decoy and the known (but withheld from training) native structure. We consider only a linear regression model versus a perceptron (that uses the sigmoid activation function), as our focus is to simply evaluate the utility of features rather than various regression methods. As Section 3 relates, we approach this lRMSD-prediction problem in two different ways, first building a model per each category (easy, medium, and hard), and then building one model for all 18 proteins (all categories combined). We scale lRMSDs to be in the range [0,1], so that sigmoid activation function in the perceptron can ensure mapping the input to an output.

Table 3, Table 4 and Table 5 relate the performance of models obtained for each category, respectively. Each of these models are trained over 2/3 of the proteins in the category they address, and tested over 1/3 of the remaining proteins in that category. Table 3 shows the (testing) performance of the linear and perceptron models trained over the 1ail, 1dtj(A), and 1wapa datasets in the easy category but tested over the rest of the datasets. Table 4 shows the testing performance of the linear and perceptron models trained over the 1bq9, 2ci2, 1hhp, 1fwp, and 1sap datasets in the medium-difficulty category but tested over the rest of the datasets. Table 5 shows the testing performance of the linear and perceptron models trained over the 1aoy, 1cc5, 2h5nd, and 2ezk datasets in the hard category byt tested over the rest of the datasets.

Table 3, Table 4 and Table 5 show that the models achieve good performance on their lRMSD-prediction task. Overall, the MSEs obtained from the models trained over dAE-learned features are lower than those trained over features obtained with PCA and Isomap. As expected, performance is the best over the easy category (see the higher regression variance).

Table 6 shows the performance of the linear regression and perceptron models trained over all categories combined. The models are trained over 2/3 of the data from each category (combined; the training datasets related above per category are combined into one training dataset) and tested over the rest of the datasets (combined).

The results in Table 6 again show good performance of the combined model (whether via linear regression or perceptron). As before, the dAE-learned features result in better performance (higher regression variance compared to PCA-obtained and Isomap-obtained features). The combined model results in lower performance than the individualized, category-specific models, suggesting that, while one combined model achieves good performance, dataset-specific models may be desirable, depending on additional information available.

## 5. Conclusions

In this paper, we investigate and evaluate AEs and AE-marginalized protein tertiary structures. A systematic evaluation points to a top-performing architecture. The utility of the learned representations is evaluated via supervised learning in discriminating between native and non-native structures.

Altogether, we believe that AEs hold great promise for the reduction and summarization of molecular structure data. Platforms such as Keras make them easy to implement, evaluate, and thus adopt, opening the way to further research on exploiting AE-featurized structures for structure–function recognition in molecular biology. Many directions of research are promising. Pursuing additional regularizations will help in further lowering the dimensionality of the loss surface. Variational AEs are also another direction of future research that can help with generating novel tertiary structures for data augmentation and other applications.

## Figures and Tables

**Table 1:** Testing dataset (* denotes proteins with a predominant β fold and a short helix). The chain extracted from a multi-chain PDB entry (shown in Column 2) to be used as the native structure is shown in parentheses. The CATH fold and architecture [41] of the known native structure is shown in Column 3. The length of the protein sequence (#aas) is shown in Column 4. The size of the Rosetta-generated decoy dataset is shown in Column 5. Column 6 shows the minimum lRMSD over decoys from the known native structure. Column 7 shows the percentage of near-native decoy structures (within some threshold lRMSD of the known native structure).

Difficulty	PDB id	CATH Fold and Architecture	# AAs	# Decoys	Min lRMSD	% Native
					(Å)	
Easy	1ail	Mainly α, Orthogonal Bundle	70	58,491	0.50	6.352
	1dtd(B)	α+β, 2-Layer Sandwich	61	58,745	0.51	22.827
	1wap(A)	Mainly β, Sandwich	68	68,000	0.60	10.192
	1tig	α+β, 2-Layer Sandwich	88	60,000	0.60	15.109
	1dtj(A)	α+β, 2-Layer Sandwich	74	60,500	0.68	22.435
Medium	1hz6(A)	α+β, Roll	64	60,000	0.72	11.325
	1c8c(A)	Mainly $\beta^{*}$, Beta Barrel	64	65,000	1.08	10.882
	2ci2	α+β, 2-Layer Sandwich	65	60,000	1.21	22.443
	1bq9	Mainly β, Single Sheet	53	61,000	1.30	1.565
	1hhp	Mainly $\beta^{*}$, Beta Barrel	99	60,000	1.52	2.486
	1fwp	α+β, 2-Layer Sandwich	69	51,724	1.56	5.819
	1sap	Mainly β, Beta Barrel	66	66,000	1.75	2.304
Hard	2h5n(D)	Mainly α, Orthogonal Bundle	123	54,795	2.00	0.845
	2ezk	Mainly α, Orthogonal Bundle	93	54,626	2.56	13.047
	1aoy	Mainly α, Orthogonal Bundle	78	57,000	3.26	10.923
	1cc5	Mainly α, Orthogonal Bundle	83	55,000	3.95	5.529
	1isu(A)	coil (Few Secondary Structures), Irregular	62	60,000	5.53	5.304
	1aly	Mainly β, Sandwich	146	53,000	8.53	2.779

**Table 2:** Comparison of MSEs of PCA, vAE, ${\mathrm{vAE}}_{\sigma_{E}}$, $dAE_{E_{PLR},D_{PLR}}$, and ${\mathrm{oAE}}_{\sigma_{E},\sigma_{D}}$ over testing datasets of target proteins; each protein is identified with the PDB id of its known native structure, with the chain shown in parentheses. Each AE architecture is trained three times, starting with initial random weights and biases, resulting in different models, whose MSE variances are shown. The mean MSE values are rounded to the second digit after the decimal sign. Higher precision is shown for the variance results, rounding to 0 all values less than $1\times 10^{-10}$.

PDB ID	PCA	vAE	${\mathrm{vAE}}_{\sigma_{E}}$	${\mathrm{oAE}}_{\sigma_{E},\sigma_{D}}$	${\mathrm{dAE}}_{E_{\mathrm{PLR}},D_{\mathrm{PLR}}}$
	Mean	Mean (Var)	Mean (Var)	Mean (Var)	Mean (Var)
1*ail*	11.69	11.69($3\times 10^{-6}$)	12.86(0)	8.73(0)	7.17(0)
1*dtd*(*B*)	11.09	11.09(0)	11.45(0)	8.21(0)	6.6(0)
1*wap*(*A*)	22.64	22.64(0)	24.12(0)	15.82($6.76\times 10^{-5}$)	12.72(0)
1*tig*	4.26	4.26(0)	5.27(0)	3.36(0)	2.41(0)
1*dtj*(*A*)	5.01	5.01($3\times 10^{-6}$)	5.54(0)	3.00(0)	2.48(0)
1*hz*6**(*A*)	3.84	3.84(0)	4.06(0)	2.29(0)	1.49(0)
1*c*8*c*(*A*)	8.35	8.35(0)	9.18(0)	4.94(0)	3.82(0)
2*ci*2	8.74	8.75(0)	9.07(0)	5.92(0)	4.53(0.026)
1*bq*9	11.08	11.08(0)	11.71(0)	8.50(0)	7.0(0)
1*hhp*	33.51	33.51(0)	35.18(0)	28.75(0)	23.69(0)
1*fwp*	10.96	10.96(0)	11.25(0)	8.42(0)	6.56(0)
1*sap*	5.03	5.03(0)	5.5(0)	3.61(0.019)	2.44(0)
2*h*5*n*(*D*)	34.21	34.22(0)	35.34(0)	28.45(0)	24.39(0.059)
2*ezk*	13.22	7.91(0)	8.54(0)	6.13($2.31\times 10^{-6}$)	5.07(0)
1*aoy*	12.82	12.82(0)	14.04(0)	8.92(0)	6.68(0)
1*cc*5	23.24	23.24(0)	23.24(0)	19.49(0)	16.5(0)
1*isu*(*A*)	21.63	21.24(0)	22.11(0)	17.66(0)	16.05(0)
1*aly*	51.98	51.98(0)	54.16(0)	47.49(0)	44.3(0)

**Table 3:** Performance of the *Easy* model. MSE and variance values are rounded to the third digit after the decimal sign.

	dAE${}_{E_{\mathrm{PLR}},D_{\mathrm{PLR}}}$		PCA		Isomap	
	Perceptron	Linear Reg.	Perceptron	Linear Reg.	Perceptron	Linear Reg.
	MSE (Var)	MSE (Var)	MSE (Var)	MSE (Var)	MSE (Var)	MSE (Var)
2D	0.025 (0.527)	0.028 (0.477)	0.029 (0.463)	0.036 (0.325)	0.038 (0.289)	0.041 (0.277)
5D	0.024 (0.559)	0.027 (0.493)	0.034 (0.360)	0.043 (0.209)	0.036 (0.180)	0.032 (0.177)
10D	0.022 (0.590)	0.026 (0.508)	0.026 (0.521)	0.035 (0.357)	0.024 (0.500)	0.033 (0.321)
20D	0.024 (0.546)	0.034 (0.370)	0.027 (0.445)	0.033 (0.391)	0.034 (0.231)	0.035 (0.219)

**Table 4:** Performance of the *Medium* model. MSE and Variance values are rounded to the third digit after the decimal sign.

	dAE${}_{E_{\mathrm{PLR}},D_{\mathrm{PLR}}}$		PCA		Isomap	
	Perceptron	Linear Reg.	Perceptron	Linear Reg.	Perceptron	Linear Reg.
	MSE (Var)	MSE (Var)	MSE (Var)	MSE (Var)	MSE (Var)	MSE (Var)
2D	0.026 (0.289)	0.030 (0.196)	0.029 (0.215)	0.032 (0.146)	0.029 (0.215)	0.032 (0.146)
5D	0.053 (0.217)	0.032 (0.152)	0.028 (0.238)	0.032 (0.031)	0.029 (0.250)	0.032 (0.011)
10D	0.025 (0.312)	0.025 (0.321)	0.028 (0.255)	0.031 (0.164)	0.031 (0.185)	0.029 (0.175)
20D	0.024 (0.351)	0.023 (0.376)	0.027 (0.222)	0.030 (0.178)	0.031 (0.201)	0.030 (0.193)

**Table 5:** Performance of the *Hard* model.MSE and Variance values are rounded to the third digit after the decimal sign.

	dAE${}_{E_{\mathrm{PLR}},D_{\mathrm{PLR}}}$		PCA		Isomap	
	Perceptron	Linear Reg.	Perceptron	Linear Reg.	Perceptron	Linear Reg.
	MSE (Var)	MSE (Var)	MSE (Var)	MSE (Var)	MSE (Var)	MSE (Var)
2D	0.017 (0.097)	0.017 (0.083)	0.017 (0.111)	0.017 (0.111)	0.019 (0.086)	0.021 (0.077)
5D	0.016 (0.139)	0.16 (0.153)	0.015 (0.184)	0.015 (0.177)	0.022 (0.180)	0.023 (0.156)
10D	0.018 (0.045)	0.016 (0.147)	0.015 (0.201)	0.015 (0.186)	0.016 (0.183)	0.017 (0.181)
20D	0.016 (0.117)	0.015 (0.185)	0.014 (0.113)	0.014 (0.102)	0.015 (0.115)	0.015 (0.093)

**Table 6:** Performance of the *Combined* model. MSE and Variance values are rounded to the third digit after the decimal sign.

	dAE${}_{E_{\mathrm{PLR}},D_{\mathrm{PLR}}}$		PCA		Isomap	
	Perceptron	Linear Reg.	Perceptron	Linear Reg.	Perceptron	Linear Reg.
	MSE (Var)	MSE (Var)	MSE (Var)	MSE (Var)	MSE (Var)	MSE (Var)
2D	0.027 (0.267)	0.032 (0.150)	0.044 (0.165)	0.043 (0.138)	0.048 (0.145)	0.040 (0.122)
5D	0.028 (0.243)	0.032 (0.151)	0.045 (0.169)	0.044 (0.138)	0.039 (0.160)	0.035 (0.112)
10D	0.028 (0.250)	0.033 (0.150)	0.043 (0.139)	0.044 (0.133)	0.040 (0.126)	0.038 (0.113)
20D	0.028 (0.248)	0.033 (0.151)	0.043 (0.041)	0.044 (0.132)	0.056 (0.021)	0.059 (0.017)

## Supplementary Materials

The following are available online.

## Abbreviations

The following abbreviations are used in this manuscript:

AE	Autoencoder
PDB	Protein Data Bank
PSP	Protein Structure Prediction
lRMSD	least Root-Mean-Squared-Deviation
MSE	Mean Squared Error
