# Supplementary material for: Evaluating Autoencoder-Based Featurization and Supervised Learning for Protein Decoy Selection
Source: Molecules. 2020 Mar 4;25(5):1146. doi: 10.3390/molecules25051146 (PMC7179114; doi:10.3390/molecules25051146)
Supplement: Supplementary file 1 [file molecules-25-01146-s001.pdf]

Supplementary Material: Evaluating Autoencoder-based Featurization and Supervised Learning for Protein Decoy Selection

Fardina Fathmiul Alam, Taseef Rahman and Amarda Shehu

Relationship of MSE to dimensionality

(a)

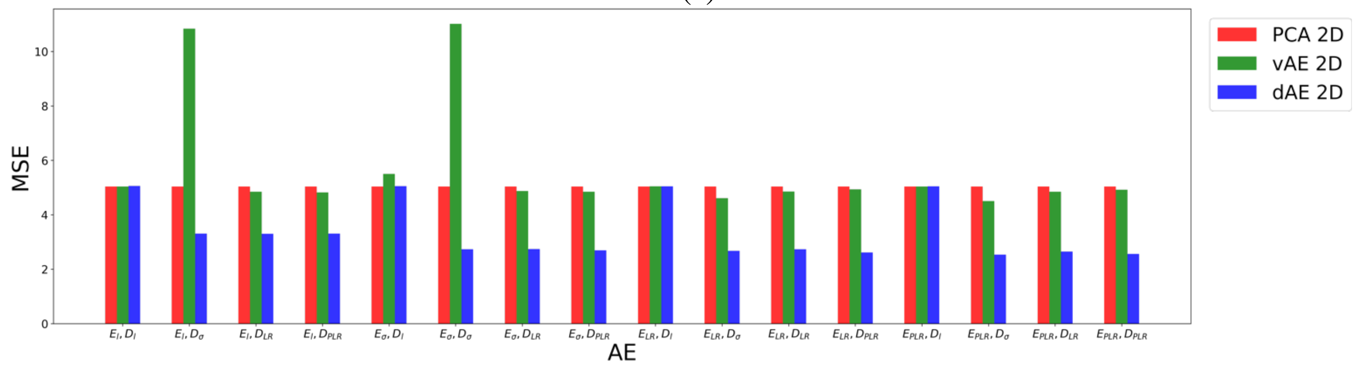

(b)

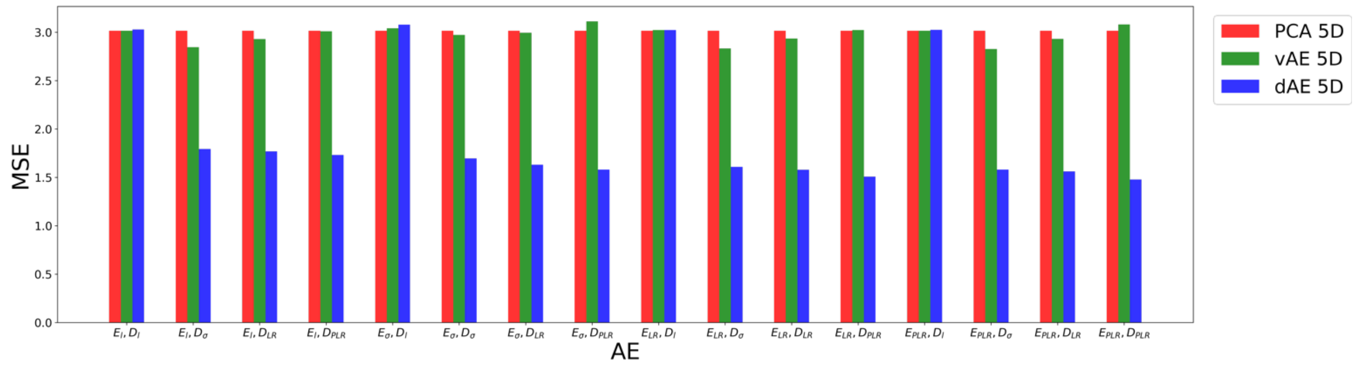

(c)

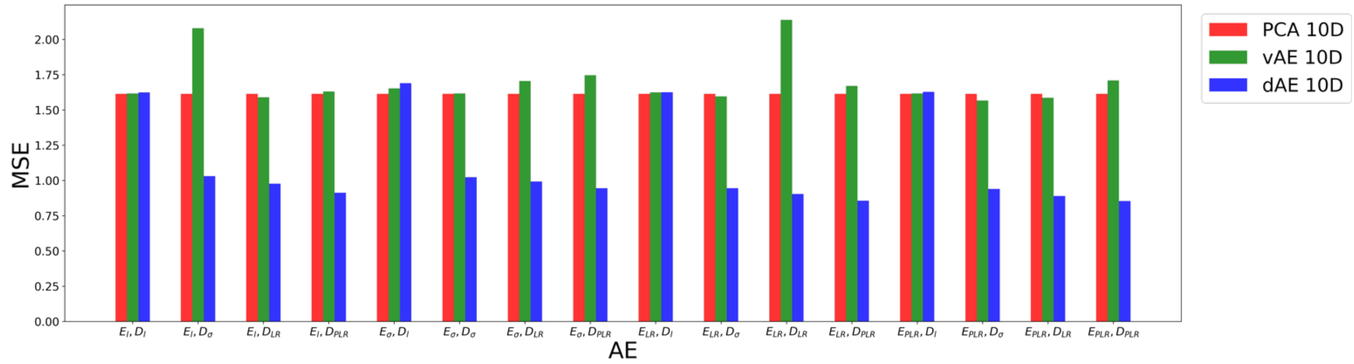

(d)

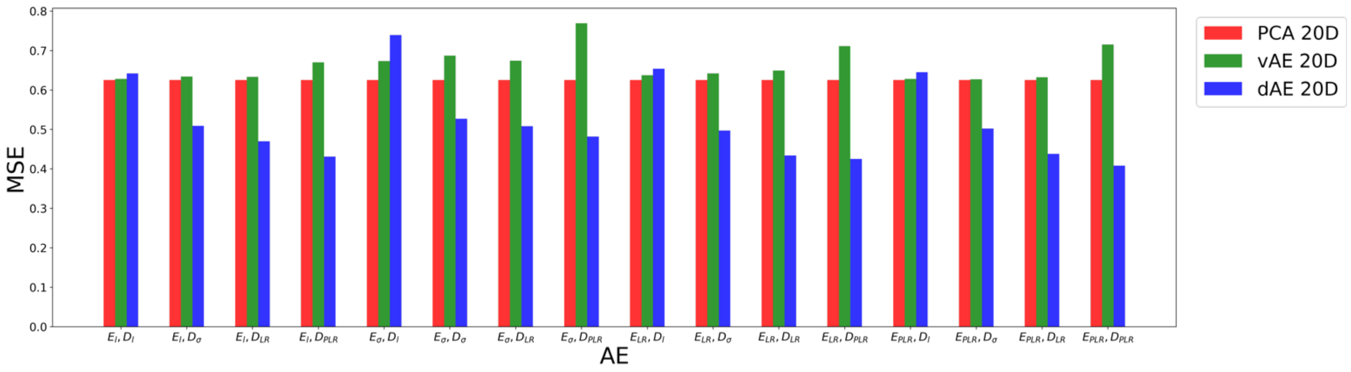

Figures 1 (a-d): Comparison of various AE architectures is related on 1DTJ(A). Rows show MSEs obtained by AE models trained to learn representations of varying dimensionality, as shown in the legends. MSEs shown on the y-axis (combinations of activation functions in encoder and decoder are listed on the x-axis) are averages over 3 runs.

(a)

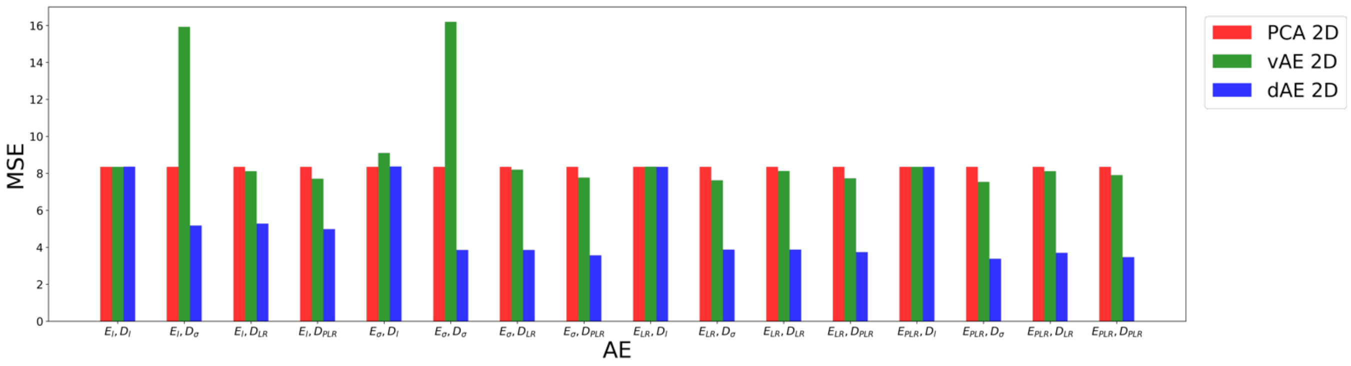

(b)

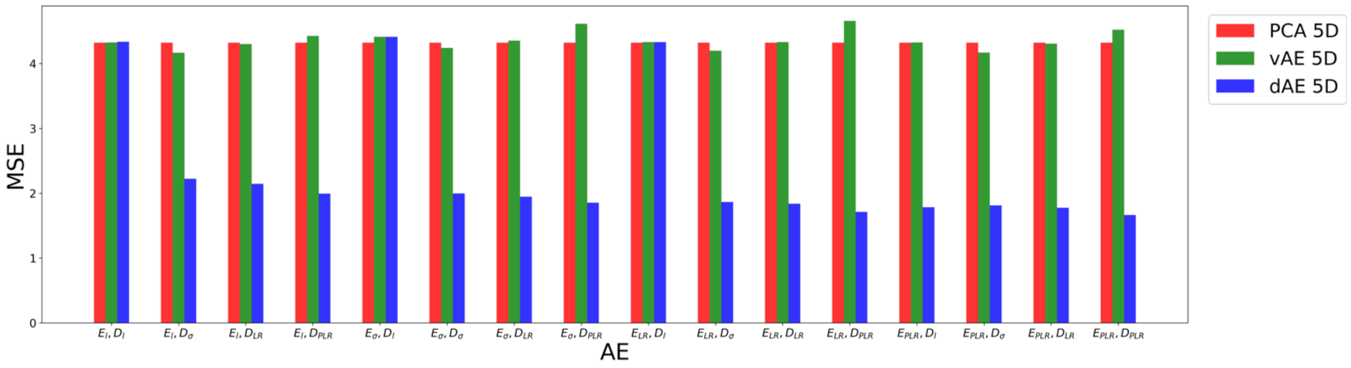

(c)

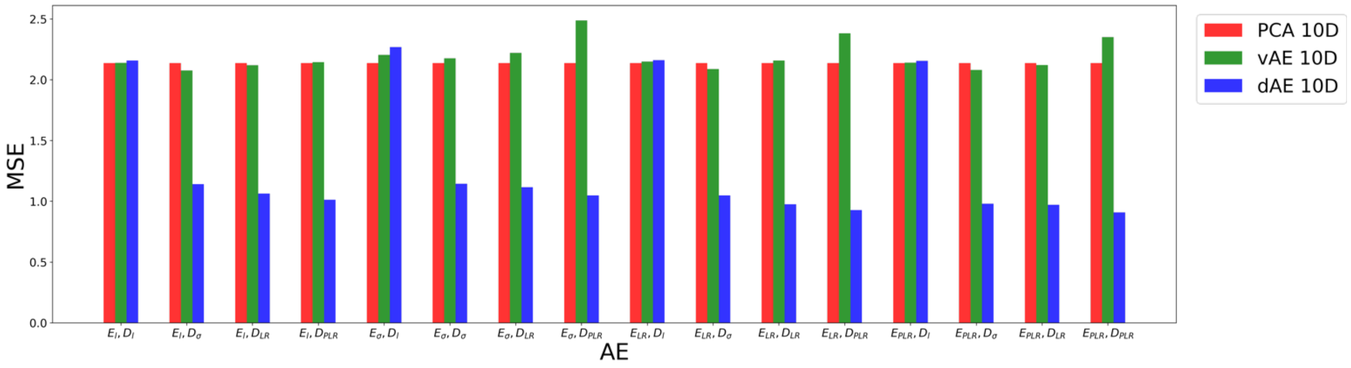

(d)

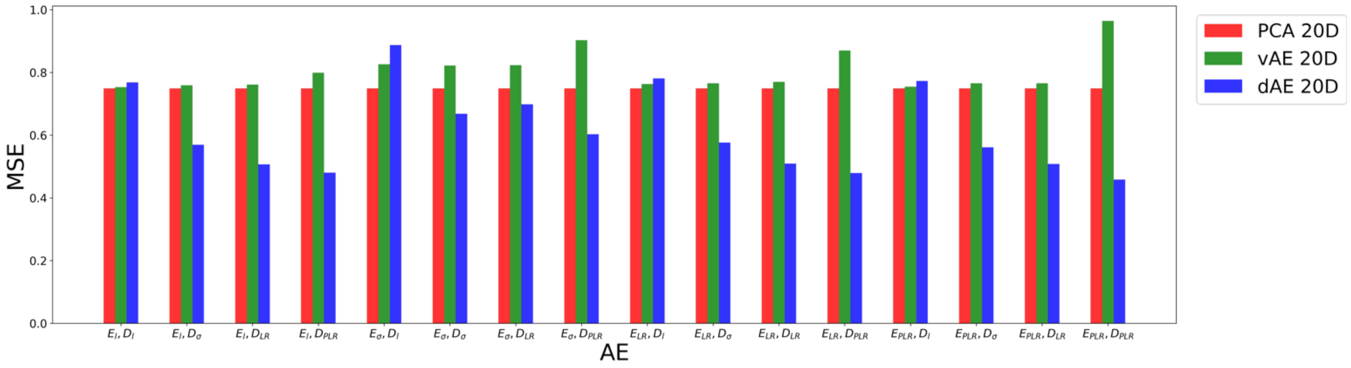

Figures 2 (a-d): Comparison of various AE architectures is related on 1C8C(A). Rows show MSEs obtained by AE models trained to learn representations of varying dimensionality, as shown in the legends. MSEs shown on the y-axis (combinations of activation functions in encoder and decoder are listed on the x-axis) are averages over 3 runs.

(a)

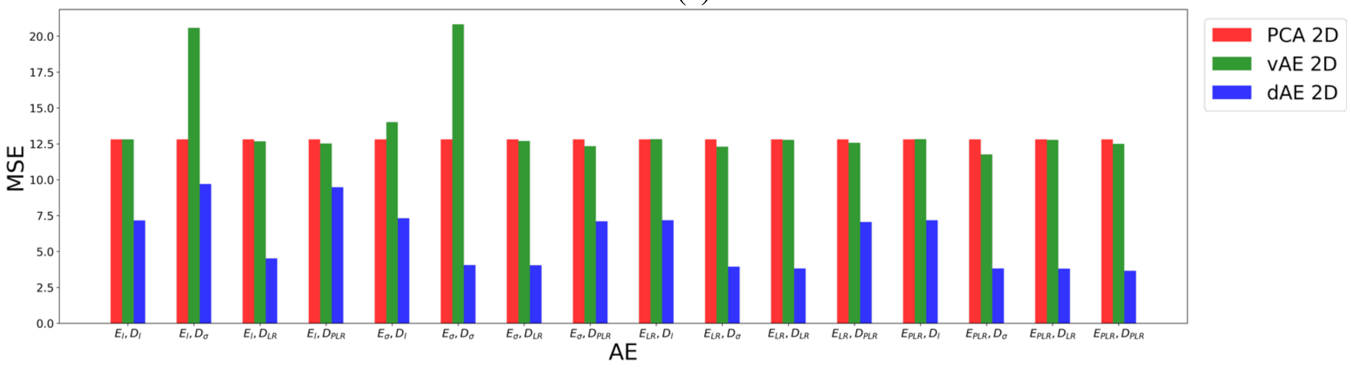

(b)

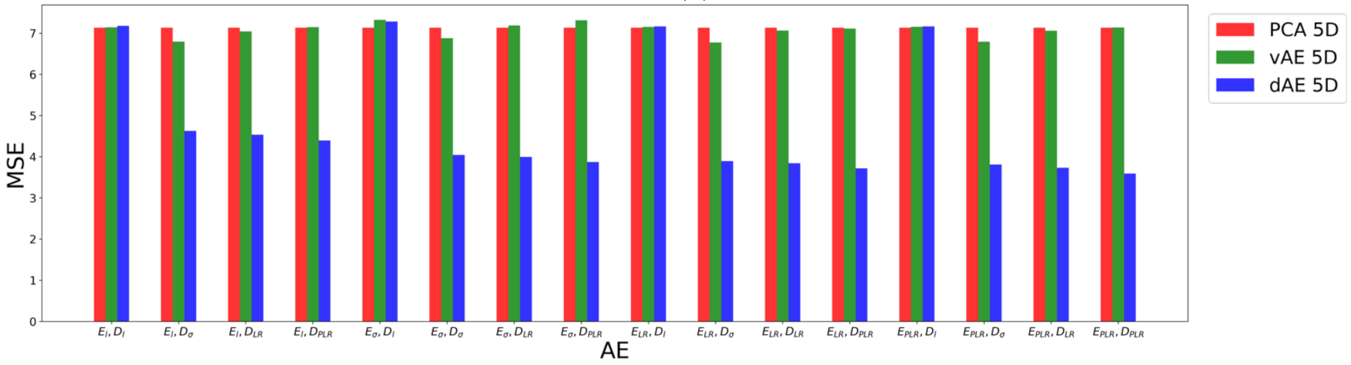

(c)

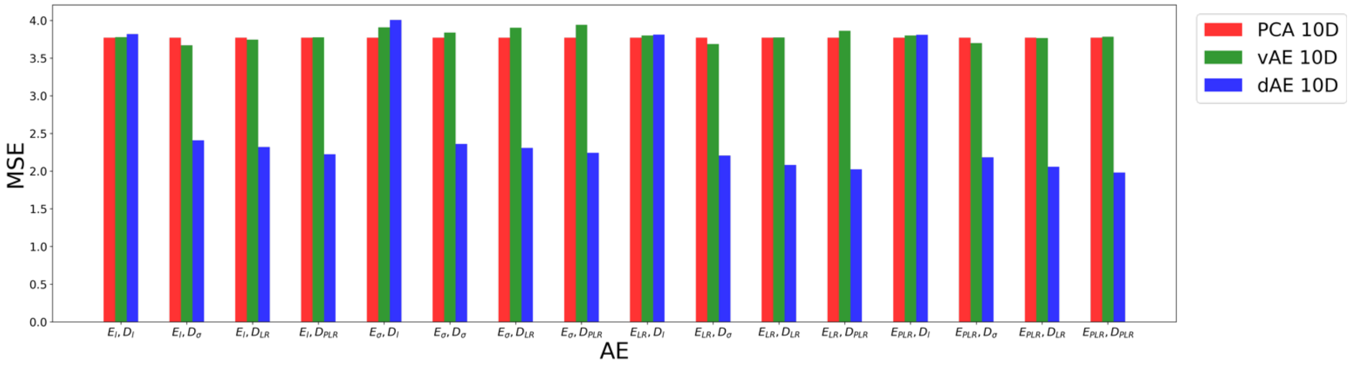

(d)

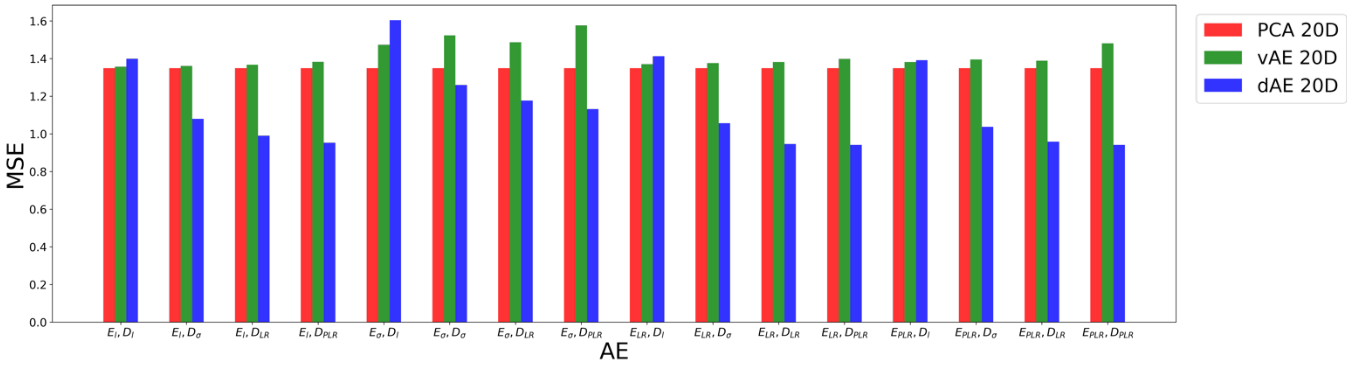

Figures 3 (a-d): Comparison of various AE architectures is related on 1AOY. Rows show MSEs obtained by AE models trained to learn representations of varying dimensionality, as shown in the legends. MSEs shown on the y-axis (combinations of activation functions in encoder and decoder are listed on the x-axis) are averages over 3 runs.

Description of Proteins

PDB ID	Protein Name
1ail	N-terminal fragment of ns1 protein from influenza a virus
1dtd(B)	Crystal structure of the complex between the leech carboxypeptidase inhibitor and the human carboxypeptidase a2 (lci-cpa2)
1wap(A)	Trp rna-binding attenuation protein in complex with l-tryptophan
1tig	Translation initiation factor 3 c-terminal domain
1dtj(A)	Crystal structure of nova-2 kh3 k-homology rna-binding domain
1hz6(A)	Crystal structures of the b1 domain of protein l from peptostreptococcus magnus with a tyrosine to tryptophan substitution
2ci2	Chymotrypsin inhibitor 2
1bq9	Rubredoxin (Formyl Methionine Mutant) from Pyrococcus Furiosus
1hhp	The three-dimensional structure of the aspartyl protease from the hiv-1 isolate bru
1fwp	Chey-binding domain of chea (residues 159-227), nmr
1sap	Hyperthermophile protein, relaxation matrix refinement structure
2h5n(D)	Crystal Structure of Protein of Unknown Function PG1108 from Porphyromonas gingivalis W83
2ezk	Solution nmr structure of the ibeta subdomain of the mu end dna binding domain of phage mu transposase, regularized mean structure
1aoy	N-terminal domain of escherichia coli arginine repressor nmr
1cc5	Crystal structure of azotobacter cytochrome c5
1isu(A)	The three-dimensional structure of the high-potential iron-sulfur protein isolated from the purple phototrophic bacterium rhodocyclus tenuis determined and refined at 1.5 angstroms resolution
1aly	Crystal structure of human cd40 ligand
